# Remote Ischaemic Preconditioning in Intra-Abdominal Cancer Surgery (RIPCa): A Pilot Randomised Controlled Trial

**DOI:** 10.3390/jcm11071770

**Published:** 2022-03-23

**Authors:** Aikaterini Papadopoulou, Matthew Dickinson, Theophilus L. Samuels, Christian Heiss, Julie Hunt, Lui Forni, Ben C. Creagh-Brown

**Affiliations:** 1 Department of Anaesthesia, King’s College Hospital, London SE5 9RS, UK; 2 Department of Clinical and Experimental Medicine, Faculty of Health and Medical Sciences, University of Surrey, Guildford GU2 7XH, UK; c.heiss@surrey.ac.uk (C.H.); j.hunt@surrey.ac.uk (J.H.); luiforni@nhs.net (L.F.); bencb@nhs.net (B.C.C.-B.); 3 Department of Anaesthesia, Royal Surrey County Hospital, Guildford GU2 7XX, UK; matthew.dickinson@nhs.net; 4 Department of Critical Care, Surrey and Sussex Healthcare NHS Trust, Redhill RH2 5RH, UK; theophilus.samuels1@nhs.net; 5 Vascular Department, Surrey and Sussex Healthcare NHS Trust, Redhill RH2 5RH, UK; 6 Department of Critical Care, Royal Surrey County Hospital, Guildford GU2 7XX, UK

**Keywords:** ischaemic preconditioning, postoperative morbidity, postoperative troponin, urinary biomarkers

## Abstract

There is limited evidence on the effect of remote ischaemic preconditioning (RIPC) following non-cardiac surgery. The aim of this study was to investigate the effect of RIPC on morbidity following intra-abdominal cancer surgery. We conducted a double blinded pilot randomised controlled trial that included 47 patients undergoing surgery for gynaecological, pancreatic and colorectal malignancies. The patients were randomized into an intervention (RIPC) or control group. RIPC was provided by intermittent inflations of an upper limb tourniquet. The primary outcome was feasibility of the study, and the main secondary outcome was postoperative morbidity including perioperative troponin change and the urinary biomarkers tissue inhibitor of metalloproteinases-2 and insulin-like growth factor-binding protein 7 (TIMP-2*IGFBP-7). The recruitment target was reached, and the protocol procedures were followed. The intervention group developed fewer surgical complications at 30 days (4.5% vs. 33%), 90 days (9.5% vs. 35%) and 6 months (11% vs. 41%) (adjusted *p* 0.033, 0.044 and 0.044, respectively). RIPC was a significant independent variable for lower overall postoperative morbidity survey (POMS) score, OR 0.79 (95% CI 0.63 to 0.99) and fewer complications at 6 months including pulmonary OR 0.2 (95% CI 0.03 to 0.92), surgical OR 0.12 (95% CI 0.007 to 0.89) and overall complications, OR 0.18 (95% CI 0.03 to 0.74). There was no difference in perioperative troponin change or TIMP2*IGFBP-7. Our pilot study suggests that RIPC may improve outcomes following intra-abdominal cancer surgery and that a larger trial would be feasible.

## 1. Introduction

Morbidity following cancer surgery may affect both short- and long-term outcomes resulting not only in prolonged hospital stay and delayed adjunct treatment but also in decreased survival and disease recurrence [1,2]. Body cavity surgery causes a significant increase in oxygen consumption and, if that remains unmet, the supply/demand imbalance can result in tissue ischaemia [3]. Although the restoration of blood flow is necessary to prevent permanent organ damage, it may cause an inflammatory response that can augment tissue injury in excess of that produced by ischemia alone [4]. Ischaemic preconditioning describes a brief episode of ischaemia that initiates a response which protects organs from sustained ischaemic events and has the potential to attenuate the ischaemic and reperfusion impact of the surgical insult. In the case of remote ischaemic preconditioning (RIPC), the stimulus is applied non-invasively via a tourniquet applied at a limb [5]. The mechanism of RIPC is not completely understood, but likely involves both neuronal and humoral factors that result in vagally mediated cardioprotection and nitric oxide-induced mitochondrial protection, respectively [6,7,8]. RIPC has been studied mostly in cardiac and vascular surgery. There is currently limited evidence about its use in general surgery, including intra-abdominal cancer surgery. The aim of the current pilot randomised controlled study was to assess the feasibility of a substantive trial and investigate whether RIPC can improve outcomes when applied to patients prior to intra-abdominal cancer surgery.

## 2. Materials and Methods

### 2.1. Study Design

This was a single-centre double-blinded interventional pilot study with randomization 1:1 into intervention (RIPC applied to an upper limb) or control (tourniquet applied but not inflated). The study was approved by the London-Fulham NRES Committee (REC reference 18/LO/1513, IRAS ID 243707, Chair Rev’d Nigel Griffin) on 26 October 2018. The protocol was registered at the CPMS (ID 38780) and ISRCTN registries (ISRCTN 11439947). A sample size of 50 participants was targeted as big enough to allow assessment of the study procedures and power analysis for a substantive trial. The study was conducted at Royal Surrey County hospital from May 2019 to April 2020. Written informed consent was obtained. A web-based randomisation system was used with block sizes of 4 and 6 and stratified per surgical specialty. The randomisation outcomes were kept in sequentially numbered opaque sealed envelopes that were opened after induction of anaesthesia. The participants, the clinical teams looking after them and the data collectors were blinded as to the whether RIPC was administered or not. The anaesthetists responsible for the intraoperative care were not blinded, as this was not practically possible.

### 2.2. Recruitment Criteria

Patients eligible to take part in the study were adults undergoing elective surgery for colorectal, pancreatic or gynaecological cancer under general anaesthesia and with an estimated morbidity risk of at least 10%, as predicted by the American College of Surgeons National Surgical Quality Improvement Project (ACS NSQIP) tool [9]. The exclusion criteria are described in the Supplemental Table S1 and included significant limb vascular or neuromuscular disease and the use of drugs that may interfere with RIPC including total intravenous anaesthesia (TIVA) with propofol.

### 2.3. Intervention

Participants allocated to the intervention group received RIPC by a member of the research team after the induction of general anaesthesia and prior to skin incision. RIPC was provided by means of inflation of an appropriately sized cuff of an automated tourniquet machine placed on an upper limb and inflated at a pressure of 200 mmHg for 5 min at a time for a total of three times with 5-minute intervals of no inflation. Participants randomized to the control group had the tourniquet placed at an upper limb but not inflated. 

### 2.4. Outcomes

The primary outcome of the study was feasibility of a randomised controlled trial. The secondary outcomes of interest were postoperative morbidity, including high sensitivity troponin I (hs-TropI) and the urinary biomarkers TIMP-2 and IGFBP-7 (NEPHROCHECK^®^), hospital length of stay and quality of life. TIMP-2 and IGFBP-7 are inducers of G1 cell cycle arrest [10]; they rise within hours of the renal insult and can be used to identify patients at risk of developing moderate to severe acute kidney injury (AKI) [11].

Blood samples for hs-TropI were collected in serum-separating tubes prior to the intervention and on the first postoperative day, and urine for the [TIMP-2]*[IGFBP-7] biomarkers was collected 4 h following the end of the operation. The samples were centrifuged, and aliquots were stored at −70 °C with the urine samples after freezing on dry ice. Following participant recruitment, the serum samples were thawed and analysed using electrochemiluminescence immunoassay (Siemens ADVIA Centaur^®^, Camberley, UK) with a hs-TropI assay range of 2.5–25,000 ng·L^−1^ and upper reference limit of 47 ng·L^−1^. The urine samples were analysed using point-of-care fluorescence immunoassay (NEPHROCHECK^®^, Astute Medical, San Diego, CA, USA) technology and the result was given as an AKIRISK™Score, calculated by the formula: AKIRISK™ Score = ([TIMP-2]*[IGFBP-7])/1000 (ng·mL^−1^)^2^. A single cut-off between 0.3 and 2 (ng·mL)^2^/1000 has been shown to be associated with at least 4 times increased risk of developing moderate-severe AKI (KDIGO stage 2–3) in the following 12 h [12]. Blood for serum creatinine was collected preoperatively and then on days 2, 5 and 7 and 6 weeks postoperatively, or when clinically indicated, and analysed immediately (Siemens Advia 1800, Camberley, UK)

Postoperative morbidity was assessed using the postoperative morbidity survey (POMS) on days 2, 3 and 5 (Supplemental Table S2) provided the participant remained in hospital and the American College of Surgeons National Safety Quality Improvement Program (ACS-NSQIP) defined complications via telephone at 30 days, 90 days and 6 months postoperatively (Supplemental Table S3). AKI was assessed using the KDIGO classification. (Supplemental Table S4). Quality of life was assessed using the EQ-5D-5L tool at the out of hospital follow-ups (Supplemental Figure S1) [13,14].

### 2.5. Statistical Analysis

The feasibility of the study included the assessment of the proportion of potentially eligible patients that were recruited to the study and followed up at the specified timepoints and the proportion of blood and urine samples collected and analysed.

Continuous variables were analysed using an unpaired Student’s *t*-test or Wilcoxon’s signed rank test as appropriate, following the Shapiro–Wilk test for normality. Categorical variables were analysed using the χ^2^ or Fisher’s exact test. Where categorical variables were assessed at three time points, the Cochran–Mantel–Haenszel χ^2^ test was used followed by groupwise analysis with time as the grouping variable and false discovery rate (fdr) adjusted *p* values. Linear and binomial regression analysis were used to assess the potential association of any independent factors with continuous and categorical outcomes, respectively, at the studied time points. The independent variables were chosen based on clinical judgement and the previous literature. The independent variables used in the binomial regression for POMS morbidities were age, American Society of Anaesthesiologists (ASA) grade, risk of overall postoperative morbidity as predicted by ACS NSQIP, treatment group, surgery and perioperative troponin change. In addition to the above, preoperative cardiovascular (CVS) disease and estimated blood loss (EBL) were included in the independent variables of the regression analysis for CVS morbidity, preoperative pulmonary disease for pulmonary morbidity and chronic kidney disease and urinary biomarkers for postoperative AKI. The independent variables used in the binomial regression for ACS NSQIP-defined complications were age, ASA grade, ACS NSQIP-predicted risk, treatment group, surgery and perioperative troponin change. Additionally, preoperative CVS disease and POMS CVS morbidity were included in the independent variables for postoperative ACS-defined CVS complications, preoperative pulmonary disease and POMS pulmonary morbidity for ACS pulmonary complications, POMS gastrointestinal morbidity for ACS gastrointestinal complications, and chronic kidney disease and Nephrocheck result for ACS urinary complications. Binomial regression for each type of POMS morbidity and ACS complication was performed with Firth’s correction. Backward elimination from the initial model was used in order to establish the best fit model. Results were considered significant if the *p* value was less than 0.05. Statistical analysis was conducted using R (version 4.0.1, Vienna, Austria) and figures were produced using the package ggplot2 [15].

## 3. Results

### 3.1. Feasibility

**Figure 1:**
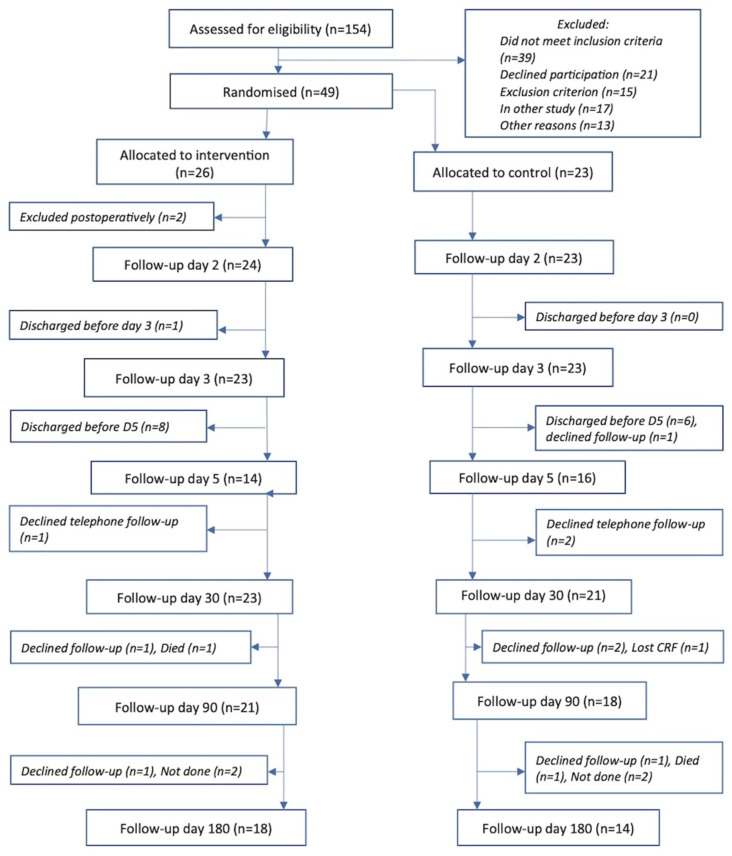
Consort diagram.

Forty-seven participants were recruited from 9 May 2019 to 10 March 2020. The study was closed early due to the COVID-19 pandemic. The reasons for exclusion from the study are described in Supplemental Table S5. The most common were estimated morbidity risk <10% and refusal to participate. Of the 47 recruited participants, 44 (94%), 39 (83%) and 32 (68%) completed the 30-day, 90-day and 6-month follow up, respectively (Figure 1).

All the randomised patients underwent the allocated treatment and there were no adverse events related to the intervention. Urine samples were collected in 40/47 (85%) of the participants and blood samples for troponin were available for all the participants preoperatively and 45/47 (96%) postoperatively. The baseline characteristics of the two groups are described in Table 1. There were no baseline imbalances.

### 3.2. In-Hospital Morbidity (POMS)

The incidence of POMS morbidity in the intervention and control groups on the different days is shown on Table 2. Groupwise analysis showed no significant difference between the two groups at any time point. Assuming that the POMS score was 0 for the discharged patients, the median [interquartile range (IQR)] overall POMS score over the first 5 postoperative days was 7 [1.75, 10.00] vs. 9 [7.50, 12.50] in the intervention vs. control group (*p* = 0.076).

**Figure 2:**
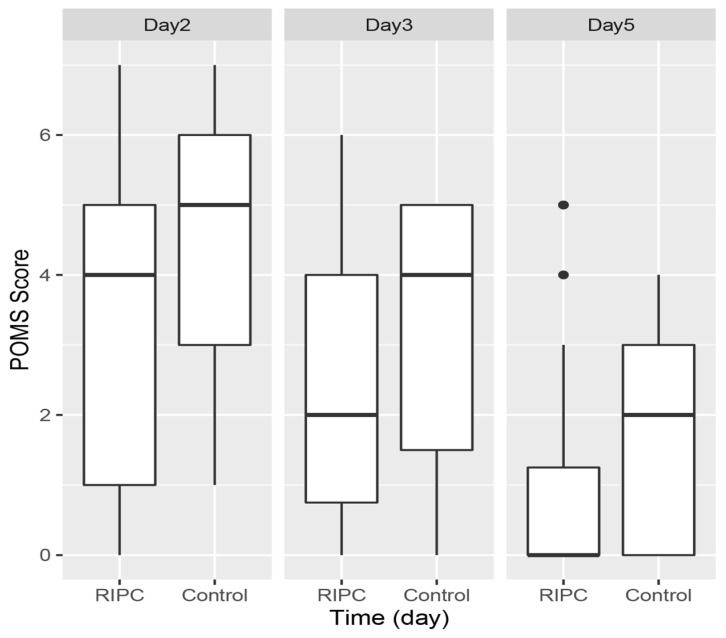
Boxplot of Postoperative Morbidity Survey (POMS) score on day 2 (4 [1, 5] vs. 5 [3, 6], adjusted *p* = 0.185), day 3 (2 [0.75, 4] vs. 4 [1.5, 5], adjusted *p* = 0.226) and day 5 (0 [0, 1.25] vs. 2 [0, 3], adjusted *p* = 0.159), in the intervention (RIPC) and control groups.

The POMS score in each treatment group on days 2, 3 and 5 is shown in Figure 2. Kruskal–Wallis test and pairwise analysis showed no difference between the treatment and intervention groups at all time points (adjusted *p* values of 0.239, 0.268 and 0.377 for days 2, 3 and 5, respectively). Mixed-effects Poisson regression of POMS score using age, ASA grade, ACS NSQIP-predicted morbidity, EBL, troponin change, treatment group, surgery and time as the random effect variables and the participants as the fixed effect variable showed that significant independent variables were treatment group, surgery and time. Being in the intervention group was associated with a lower POMS score, OR 0.79 (95% CI 0.63 to 0.99), whereas gynaecological and pancreatic surgery were associated with higher POMS scores, OR 2.22 (95% CI 1.61 to 3.03) for gynaecological surgery and OR 2.76 (95% CI 1.97 to 3.85) for pancreatic surgery. Using the overall POMS mean values with a type I error of 0.05 and power of 80%, a sample size of 116 participants would be required for a study to show a significant change in overall POMS. To allow for up to 20% dropout, 140 participants should be recruited.

Overall CVS POMS morbidity was 13/24 (54%) vs. 17/22 (77%) in the intervention vs. control groups (*p* = 0.129) and there was no difference between the two groups at any time point (*p* = 0.13). Binomial regression for any CVS morbidity showed that surgery was the only significant factor. The OR for gynaecological surgery was 32.62 (95% CI 5.33 to 407.73) and for pancreatic surgery, it was 24.69 (95% CI 3.09 to 30.68). Overall preoperative hs-TropI was median [IQR] 4 ng·L^−1^ [3, 8] and postoperative hs-TropI was 6 ng·L^−1^ [3, 10] (*p* = 0.186). Only one patient had postoperative hs-TropI above the upper reference limit; however, the patient’s preoperative hs-TropI was also above the upper reference limit. There was no difference in the perioperative troponin change between the intervention and control groups, median [IQR] 1 [0.96, 1.58] vs. 1 [1, 1.38], respectively (*p* = 0.617). Linear regression analysis of perioperative troponin change with independent variables age, preoperative CVS disease, ASA grade, ACS NSQIP predicted morbidity, EBL and surgery showed that age and surgery were the significant variables, with gynaecological and pancreatic surgery and increasing age being associated with greater troponin change, with odds ratio (OR) of 2.29 (95% CI 1.40 to 3.75), 2.73 (95% CI 1.58 to 4.72) and 1.04 (95% CI 1.01 to 1.06), respectively.

There was significantly less renal morbidity in the intervention group on day 5 (adjusted *p* = 0.006). However, further analysis of the type of morbidity showed that the difference was in the presence of urinary catheter rather than the development of AKI on day 5. The overall incidence of AKI was 7/47 (15%) in hospital and 9/47 (19%) within 6 weeks postoperatively. Four of these patients underwent colorectal surgery, one gynaecological and four pancreatic. Four of the 24 patients (16.67%) in the intervention group and five of the 23 in the control group (21.7%) developed AKI (*p* = 0.724). Of the seven in-hospital cases of AKI, five developed in the first 24 h, one at 24–48 h and one on day 7. Logistic regression of in-hospital AKI showed that troponin change was significant, OR = 12.29 (95% CI 1.82 to 219.70). TIMP-2*IGFBP-7 at 4 h postoperatively was 0.56 [0.37, 0.99] vs. 0.21 [0.12, 0.42] in patients with and without AKI in the following 12–24 h (*p* = 0.213). However, four of the five (80%) cases of AKI in the first 24 h were stage 1 and only one (20%) was stage 2. There was no difference in TIMP-2*IGFBP-7 values between the treatment groups, 0.19 [0.11, 0.31] vs. 0.34 [0.15, 0.76], intervention vs. control, (*p* = 0.1016). TIMP-2*IGFBP-7 was ≥0.3 in 5/20 (25%) vs. 12/20 (60%) of patients in the intervention and control groups, respectively (*p* = 0.054). 

The overall incidence of pulmonary POMS morbidity was 10/24 (42%) vs. 14/22 (64%) in the intervention vs. control groups (*p* = 0.232). Logistic regression of any POMS pulmonary morbidity showed that only surgery was significant with an OR of 25.62 (95% CI 3.41 to 366.87) for pancreatic surgery and 7.1 (95% CI 1.46 to 48.04) for gynaecological surgery.

The overall incidence of POMS gastrointestinal morbidity was 15/24 (63%) vs. 17/22 (77%) in the intervention vs. control group (*p* = 0.346). Logistic regression for any gastrointestinal morbidity showed that only surgery was significant with an OR of 10.03 (95% CI 1.68 to 110.71) for pancreatic and 3.69 (95% CI 0.93 to 16.11) for gynaecological surgery.

Overall pain morbidity was 14/24 (58%) vs. 17/22 (77%) in the intervention vs. control group (*p* = 0.217). The treatment group was not an independent predictor of pain morbidity in univariate analysis. The incidence of other types of POMS morbidity was overall small and is described in Table 2.

### 3.3. Length of Stay

Length of stay was 5 [3.5 to 8.5] vs. 7 [4.25 to 8] in the intervention vs. control group, *p* = 0.544. Linear regression with independent variables age, ASA, NSQIP, treatment group and surgery showed that only the overall POMS score was a significant independent variable, OR 1.52 (95% CI 1.07 to 2.16).

### 3.4. Out of Hospital Complications (ACS NSQIP)

**Figure 3:**
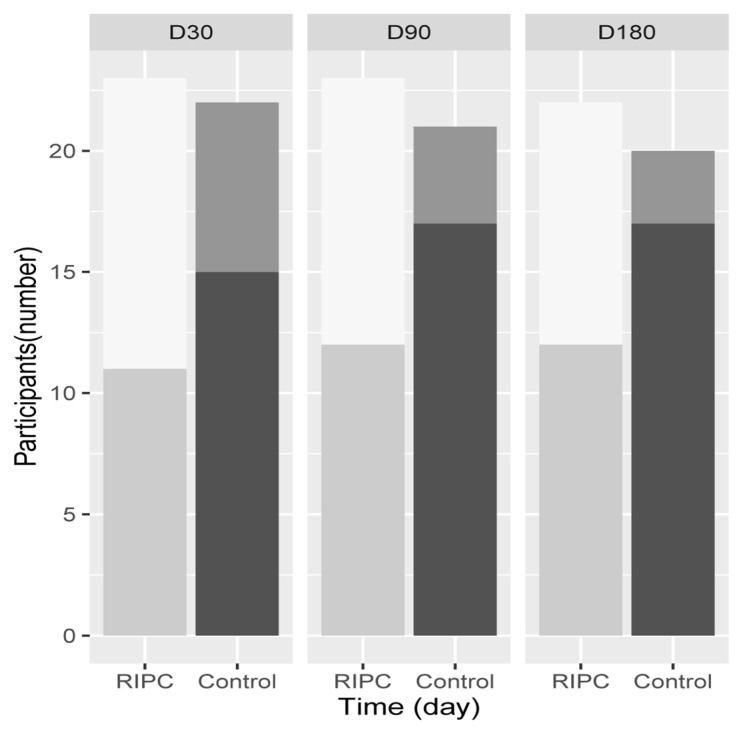
Barplot of postoperative complications (American College of Surgeons) at day 30 (11/23 (47.82%) vs. 15/22 (68.18%)), day 90 (12/23 (52.17%) vs. 17/21 (80.95%)) and day 180 (12/22 (54.55% vs. 17/20 (85%)), *p* = 0.003 in the intervention (RIPC) and control groups, respectively. 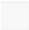 RIPC group without morbidity, 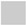 RIPC group with morbidity, 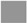 Control group without morbidity, 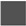 Control group with morbidity.

The incidence of any ACS complication is shown in Figure 3 and described in detail in Table 3.

Groupwise analysis did not show a difference between the two groups (adjusted *p* of 0.165, 0.062 and 0.062 at 30 days, 90 days and 6 months, respectively). Logistic regression of any ACS postoperative complication showed that the treatment group was significant at day 90 and 6 months with an OR of 0.28 (95% CI 0.07 to 0.99) and 0.18 (95% CI 0.03 to 0.74), respectively.

There was no difference in the incidence of cardiovascular complications between the two groups (*p* = 0.646). Logistic regression for day 30 CVS complications showed that the presence of in-hospital POMS CVS morbidity was significant, OR 11.63 (1.54 to 221). For day 90 and 180, preoperative cardiovascular morbidity was significant, OR 5.13 (95% CI 1.18 to 26.15) and OR 6.4 (95% CI 1.3 to 40.78), respectively.

There was no difference in the incidence of urinary complications between the two groups (*p* = 0.947). Within 6 months in the intervention vs. control group, 3/18 (16.67%) vs. 2/14 (14.29%) patients were treated for a urinary tract infection, 4/24 (16.67%) vs. 5/23 (21.74%) had AKI and 0 vs. 1/15 needed renal replacement therapy. Logistic regression of urinary complications showed that for 30-day and 90-day complications, troponin change was significant with OR 6.29 (95% CI 1.06 to 61.2) and 4.457 (95% CI 1.2 to 22.01), respectively, whereas for 6-month complications, the ACS NSQIP predicted morbidity was significant with an OR of 1.24 (95% CI 1.09 to 1.49).

Groupwise analysis of pulmonary complications showed no significant difference. Logistic regression showed that for day 180, the treatment group was the only significant factor, with the RIPC group having an OR of 0.2 (95% CI 0.03 to 0.92). No significant variables were identified for day 90 (Group *p* 0.09) or day 30. 

Groupwise analysis of gastrointestinal complications showed no difference between the treatment groups at any time point. Logistic regression for day 30 showed that in-hospital POMS gastrointestinal morbidity was significant at all time points, OR 6.27 (95% CI 1.22 to 51.57), 6.3 (95% CI 1.23 to 51.72) and 7.15 (95% CI 1.41 to 53.65) at day 30, 90 and 6 months, respectively. Further logistic regression with independent variables POMS gastrointestinal morbidity on days 2, 3 and 5 showed that only morbidity on day 5 was significant, with OR 6.48 (95% CI 1.48 to 33.77) at day 30, 5.963 (95% CI 1.35 to 31.28) at day 90 and 7.93 (95% CI 1.47 to 57.62) at day 180.

Surgical complications developed in 4.5% vs. 33% at 30 days, 9.5% vs. 35% at 90 days and 11% vs. 41% at 6 months in the intervention vs. control group (*p* = 0.0006). Groupwise analysis showed that the difference was significant at all time points (adjusted *p* of 0.033, 0.044 and 0.044 at day 30, 90 and 180, respectively). Logistic regression showed that the treatment group was a significant independent variable, OR 0.04 (95% CI 0.001, 0.34), 0.12 (0.02, 0.65) and 0.12 (95% CI 0.007, 0.89) at 30 days, 90 days and 6 months, respectively. Additionally, hs-TropI change was a significant predictor of 6-month complications, OR 7.14 (95% CI 1.16 to 408.54).

## 4. Discussion

We performed a pilot randomised controlled trial including 47 patients on the use of RIPC in intra-abdominal cancer surgery. Approximately 30% of the potential participants were recruited in the study, the relatively low proportion being mostly due to a significant number of patients having a predicted risk for postoperative morbidity <10%. All the randomised patients underwent the allocated treatment, and at least 85% had blood and urine samples analysed. The protocol procedures were followed and, although up to a third of recruited participants were lost to follow up at 6 months, the incidence did not differ per treatment allocation group. We collected data to assist with power analysis and concluded that a larger study with 80% power to detect a significant difference in overall POMS score would be feasible and would require 140 participants. Given the rate of recruitment of our study, a powered substantive trial would likely require multicentre recruitment.

In our study, the intervention group had significantly less POMS renal morbidity on day 5 and fewer surgical complications at 30 days, 90 days and 6 months postoperatively. Additionally, being in the intervention group was a significant independent variable for lower overall POMS score, fewer pulmonary complications at 6 months, fewer surgical complications at all time points and fewer overall ACS NSQIP-defined complications at 90 days and 6 months. There was, however, no significant association between the treatment group and either cardiovascular morbidity including perioperative troponin change or the renal stress biomarker TIMP-2*IGFBP-7.

Despite the significant difference detected between the two groups at POMS day 5 renal morbidity, further analysis of the type of morbidity showed that the main difference was in the presence of a urinary catheter rather than the development of AKI on day 5. The significance of this finding is unclear and unlikely to indicate a renoprotective effect of RIPC; it could, however, suggest a greater concern of the responsible clinical team for the overall progress of the patient. The use of RIPC has been associated with reduced incidence of AKI both in cardiac and non-cardiac surgery [16,17]. A meta-analysis of the use of RIPC in cardiac surgery by Deferrari et al. concluded that RIPC reduced the risk of AKI in patients with volatile maintenance of anaesthesia (OR 0.57, 95% CI 0.41 to 0.79) [18]. Zarbock et al. suggested that the failure of other studies to show significant benefit is likely due to the preferential beneficial effect of RIPC on high-risk patients [19]. It is possible that, despite the use of a predicted ACS NSQIP risk score of at least 10% as an inclusion criterion, our patient cohort was not of high enough risk to exhibit a significant reduction in either cardiovascular morbidity or AKI following RIPC. This is supported by the low levels of postoperative hsTrop-I in our patient cohort that were well below the upper reference limit, as well as the low incidence of AKI stages 2–3.

In our study, being in the intervention group was a significant independent variable for lower overall POMS score (as assessed on days 2, 3 and 5), which, in turn, was the only variable associated with prolonged length of stay. This finding is in line with previous studies that showed that POMS morbidity on postoperative days 3 and 5 after major surgery is associated with a longer hospital stay [20,21].

Amongst the surgical complications, the greatest difference between the two groups was observed in the incidence of anastomotic leak. No studies investigating the effect of RIPC on postoperative leak following bowel anastomosis were identified; however, a meta-analysis of gastric ischaemic preconditioning prior to oesophagectomy showed that the intervention reduces the incidence of severe anastomotic leak that requires reoperation (OR 0.27, 95% CI 0.14 to 0.5) [22]. Animal studies have been inconclusive so far, with suggestions of better histopathologic findings of mucosal injury and anastomotic healing but no difference in anastomotic bursting pressures compared to the control group [23,24].

Regarding the postoperative pulmonary complications, the greatest difference between the two groups was noted in the incidence of pneumonia and respiratory failure. A study of RIPC in a rat cardiopulmonary bypass model showed that RIPC attenuated postoperative lung injury, as indicated by lower protein content in the bronchoalveolar lavage fluid, less severe alveolar wall thickening, reduced neutrophil infiltration and increased dynamic lung compliance. The anti-inflammatory cytokines interleukin-4 (IL-4) and IL-10 were found to be significantly higher in the serum of the RIPC compared to the control group, potentially indicating that the pulmonoprotective effects of RIPC are due to its anti-inflammatory effects [25].

Perioperative hsTrop-I change in our study was an independent predictor of postoperative AKI, 30- and 90-day urinary complications and 6-month surgical complications. This outcome is in keeping with Noordzij’s study that showed that troponin rise following abdominal surgery, in addition to its association with increased mortality, also had an increased risk of non-cardiac complications such as sepsis, anastomotic leak, respiratory insufficiency, wound infection and bleeding [26].

Our study adds further evidence on the use of RIPC in non-cardiac non-vascular surgery and is the first in our knowledge to study the effect of RIPC on TIMP-2*IGFBP-7 and POMS morbidity in this setting. One of its limitations is that, as a pilot study, it has not been powered to detect a difference between the intervention and control groups. Additionally, we assumed that the POMS on day 5 is zero for patients discharged prior to that day and our out-of-hospital assessment of morbidity has been cumulative over time, which, in combination with 30% of participants being lost at the 6-month follow up, may have overestimated the incidence of 6-month complications. Although the study aimed to recruit patients above a certain threshold of predicted morbidity undergoing abdominal surgery, each type of surgery is associated with its own specific potential complications and, given our relatively small sample size, our results should be interpreted with caution.

In conclusion, our pilot study suggests that RIPC may limit postoperative morbidity in patients undergoing intra-abdominal cancer surgery and, given that RIPC is a simple, non-invasive and safe intervention, we recommend a definite suitably sized clinical trial.

## Figures and Tables

**Table 1:** Baseline characteristics of participants in the remote ischaemic preconditioning (RIPC) and control groups.

Participant Characteristics	RIPC (Number (%) or Median [IQR]), *n* = 24	Control (Number (%) or Median [IQR]), *n* = 23	*p*-Value
Age (years)	73 [63, 77]	72 [61, 78]	0.647
ASA 3	16 (67%)	11 (48%)	0.312
Hypertension	12 (52%)	11(52%)	>0.999
Ischaemic heart disease	2 (8%)	2 (9%)	>0.999
TIA/Stroke	3 (13%)	1 (4%)	0.609
Asthma/COPD	2 (8%)	1 (4%)	>0.999
CKD	4 (17%)	6 (26%)	0.494
Diabetes	5 (22%)	3 (14%)	0.701
Smoking	3 (13%)	3 (14%)	>0.999
Obesity	12 (52%)	16 (76%)	0.180
Underweight	1 (4%)	2 (10%)	0.599
Functionally dependent	1 (4%)	1 (5%)	>0.999
Haemoglobin (g·dL^−1^)	122 [113, 136]	118 [110, 131]	0.225
ACS-NSQIP	17 [13, 24]	21 [13, 25]	0.742
Eq-5d-5l score	0.889 [0.813, 1]	0.922 [0.814, 1]	0.852
Health (Visual Analogue Scale)	78 [53, 80]	80 [69, 88]	0.284
Colorectal cancer surgery	8 (33%)	7 (30%)	
Gynaecological cancer surgery	10 (42%)	10 (44%)	
Pancreatic cancer surgery	6 (25%)	6 (26%)	
Total	24	23	

ASA, American society of anaesthesiologists; TIA, transient ischaemic attack; COPD, chronic obstructive pulmonary disease; CKD, Chronic kidney disease; ACS-NSQIP, American College of Surgeons—National surgical quality improvement project.

**Table 2:** Postoperative Morbidity Survey (POMS) on days 2, 3 and 5 in the remote ischaemic preconditioning (RIPC) and the Control group. The *p* values are derived from Cochran–Mantel–Haenszel tests comparing the development of complications between the intervention and control groups stratified by time.

POMS Morbidity	Day 2 RIPC	Day 2 Control	Day 3 RIPC	Day 3 Control	Day 5 RIPC	Day 5 Control	*p*
Cardiovascular	12/24 (50%)	16/23 (69.57%)	7/23 (30.43%)	9/23 (39.13%)	2/14 (14.29%)	5/17 (29.41%)	0.133
Pulmonary	10/24 (41.67%)	14/23 (60.87%)	9/23 (39.13 %)	9/23 (39.13%)	3/14 (21.43%)	4/17 (23.53%)	0.475
Gastrointestinal	13/24 (54.17%)	15/23 (65.22%)	10/23 (43.48%)	11/23 (47.83%)	9/14 (64.29%	5/17 (29.415)	0.892
Renal	21/24 (87.5%)	23/23 (100%)	17/23 (73.91%)	19/22 (86.36%)	4/11 (36.36%)	14/17 (82.35%)	0.002
Pain	14/24 (58%)	17/23 (74%)	11/23 (48%)	17/22 (77%)	6/14 (43%)	5/17 (29%)	0.179
Wound	1/24 (4.2%)	2/23 (8.7%)	0/23	1/23 (4.3%)	0/14	1/17 (5.9%)	0.372
Haematological	1/24 (4.2%)	2/23 (8.7%)	0/23	3/23 (13%)	0/14	1/17 (5.9%)	0.126
Infectious	8/24 (33.33%)	9/23 (39.13%)	5/23 (21.74%)	5/22 (22.73%)	4/14 (28.57%)	5/17 (29.41%)	0.888
Any POMS morbidity	22/24 (92%)	23/23 (100%)	18/23 (78%)	22/23 (96%)	11/14 (79%)	16/17 (94%)	0.026

**Table 3:** Postoperative complications (American College of Surgeons) at 30 days, 90 days and 6 months postoperatively in the remote ischaemic preconditioning (RIPC) and control groups. The *p* values are derived from Cochran–Mantel–Haenszel tests comparing the development of complications between the intervention and control groups stratified by time (categorical outcomes) or Kruskal–Wallis one way analysis of variance (numerical outcomes).

American College of Surgeons (ACS) Complications	Day 30 RIPC	Day 30 Control	Day 90 RIPC	Day 90 Control	Day 180 RIPC	Day 180 Control	*p*
Cardiovascular	3/23 (13%)	3/21 (14.29%)	5/23 (21.74%)	5/18 (27.78%)	5/22 (22.73%)	5/16 31.25%)	0.646
Respiratory	1/22 (4.5%)	4/21 (19%)	2/21 (9.5%)	6/19 (31.57%)	2/19 (10.53%)	7/17 (41.18%)	0.0046
Gastrointestinal	6/23 (26%)	9/21 (42.86%)	6/23 (26%)	9/20 (45%)	6/20 (30%)	9/18 (50%)	0.052
Urinary	7/23 (30.43%)	5/21 (23.8%)	7/22 (31.82%)	7/19 (36.84%)	7/19 (36.84%)	7/17 (41.18%)	0.947
Surgical	1/23 (4.3%)	7/21 (33.33%)	2/21 (9.5%)	7/20 (35%)	2/18 (11.11%)	7/17 (41.18%)	0.0006
Haematological	5/23 (21.74%)	9/20 (45%)	5/23 (21.74%)	9/19 (47.37%)	5/21 (23.8%)	9/17 (52.94%)	0.005
Health Visual Analogue Scale	72 [54, 83]	80 [70, 85]	80 [75, 90]	80 [75, 84]	75 [60, 90]	80 [70, 80]	0.875
Quality of life (EQ-5D-5L)	0.931 [0.668, 1]	0.838 [0.788, 0.866]	0.930 [0.800, 0.963]	0.916 [0.824, 1]	0.874 [0.751, 1]	0.933 [0.861, 1]	0.343

## Data Availability

The data are available from the corresponding author upon reasonable request.

## Supplementary Materials

The following supporting information can be downloaded at: https://www.mdpi.com/article/10.3390/jcm11071770/s1, Supplemental Table S1. RIPCa exclusion criteria, Supplemental Table S2. Postoperative Morbidity Survey (POMS) definitions, Supplemental Table S3. ACS NSQIP definitions of postoperative complications, Supplemental Table S4. KDIGO AKI classification, Supplemental Figure S1. EQ-5D-5L quality of life questionnaire, Supplemental Table S5. Reasons for exclusion from the RIPCa study.
