# Supplementary material for: Remote Ischaemic Preconditioning in Intra-Abdominal Cancer Surgery (RIPCa): A Pilot Randomised Controlled Trial
Source: J Clin Med. 2022 Mar 23;11(7):1770. doi: 10.3390/jcm11071770 (PMC8999621; doi:10.3390/jcm11071770)
Supplement: Supplementary file 1 [file jcm-11-01770-s001.zip › jcm-1626253-supplementary.pdf]

Journal of
Clinical Medicine

MDPI

Table S1. RIPCa exclusion criteria.

1	Patients unable to give consent
2	Emergency surgery
3	Total intravenous anaesthesia
4	Pregnancy
5	Recent (< 1 month) or ongoing acute myocardial infarction
6	Unstable or ongoing angina
7	Peripheral vascular disease: any previous intervention for peripheral arterial insufficiency, or any reason to clinically suspect arterial insufficiency such as history of claudication
8	History of vascular intervention in the limb to be used for RIPC
9	Thromboembolic disease: recent (<1 month) DVT or any previous unprovoked DVT
10	Significant coagulopathy (INR>3.0 or PLT<25) or bleeding diathesis
11	Sickle cell disease
12	Neuromuscular diseases including Multiple Sclerosis, Myasthenia Gravis, Motor Neuron disease, or any congenital neurological condition
13	Use of sulfonylureas or nicorandil

Table S2. Postoperative Morbidity Survey (POMS) definitions.

Morbidity type	Criteria
**Cardiovascular**	Diagnostic tests or therapy within the last 24 for any of the following: de novo myocardial infarction or ischaemia, hypotension (requiring pharmacological therapy or fluid therapy >200 mL/h), atrial or ventricular arrhythmias or cardiogenic pulmonary oedema.
**Neurological**	Presence of a de novo focal deficit, coma or confusion/delirium
**Pulmonary**	De novo requirement for supplemental oxygen or other respiratory support (e.g. mechanical ventilation or CPAP)
**Renal**	Presence of oliguria (<500 mL/day), increased serum creatinine (>30% from preoperatively) or urinary catheter in place
**Wound**	Wound dehiscence requiring surgical exploration, drainage of pus from the operation wound, wound ooze or swab taken in the past 24h
**Gastrointestinal**	Unable to tolerate enteral diet (either by mouth or via a feeding tube) for any reason including nausea, vomiting and abdominal distension.
**Pain**	Surgical wound pain significant enough to require parenteral opioids or regional analgesia
**Haematological**	Requirements for any of the following within the last 24 hours: packed erythrocytes, platelets, fresh frozen plasma or cryoprecipitate
**Infectious**	Currently on antibiotics or temperature >38 ^{o}C in the last 24 hours

Table S3. ACS NSQIP definitions of postoperative complications.

**Medical complications**		
Neurological	Acute cerebrovascular event:	
	TIA	A transient episode of neurologic dysfunction without acute infarction or in the absence of appropriate imaging as lasting for < 60 minutes
	Stroke	An acute embolic, thrombotic or haemorrhagic event with persistent motor, sensory or cognitive dysfunction.
	Confusion or altered mental status	As documented in the medical notes or assessed by the Glasgow Coma Scale.
Cardiovascular	Cardiac arrest	Cardiac arrest documented in the medical notes
	Acute myocardial infarction	An increase in serum troponin above the 99th percentile upper reference limit and at least one of the following criteria: symptoms of ischaemia; new or presumed new significant ST segment or T wave ECG changes or new left bundle branch block; development of pathological Q waves on ECG; radiological or echocardiographic evidence of new loss of viable myocardium or new regional wall motion abnormality; identification of an intracoronary thrombus at angiography or autopsy
	New arrhythmia	ECG evidence of new cardiac rhythm disturbance.
	New or worsened heart failure	New or worsening symptoms of heart failure (e.g. dyspnea) and/or radiographic evidence of pulmonary congestion or echocardiographic evidence of acutely impaired or worsened right or left ventricular function.
	Deep vein thrombosis	A new blood clot or thrombus within the venous system diagnosed by appropriate imaging such as ultrasound or CT.
	Pulmonary embolism	A new blood clot or thrombus within the pulmonary arterial system diagnosed by scintigraphy or CT angiography or echocardiography.
Respiratory	Pneumonia	Two or more serial chest radiographs (or one radiograph for patients with no underlying pulmonary or cardiac disease) with at least one of the following:<br>(1) new or progressive and persistent infiltrates<br>(2) consolidation<br>(3) cavitation<br><br>and at least one of the following:<br>(1) fever (>38°C) with no other recognised cause<br>(2) leucopaenia (white cell count < 4 x 10^9 l^{-1}) or leucocytosis (white cell count >12 x 10^9 l^{-1})<br>(3) for adults >70 years old, altered mental status with no other recognised cause;<br><br>and at least two of the following:

		(1) new onset of purulent sputum or change in character of sputum, or increased respiratory secretions, or increased suctioning requirements<br>(2) new onset or worsening cough, or dyspnoea, or tachypnoea<br>(3) rales or bronchial breath sounds<br>(4) worsening gas exchange (hypoxaemia, increased oxygen requirement, increased ventilator demand).
	Respiratory failure	Postoperative PO2 <8kPa on room air or a PaO2;FiO2 ratio<40 kPa or arterial oxyhaemoglobin saturation measured with pulse oximetry<90% and requiring oxygen therapy.
	Bronchospasm	Newly detected expiratory wheezing treated with bronchodilators.
Gastrointestinal	Paralytic ileus	Failure to tolerate solid food or defecate for three or more days after surgery.
	Ischaemic colitis	As diagnosed by CT scan, endoscopy or reoperation
	Gastrointestinal bleed	Clinical or endoscopic evidence of blood in the gastrointestinal tract
Urinary	Urinary tract infection	A positive urine culture of $>10^5$/mL colony forming units with no more than 2 species of micro-organisms and with at least one of the following symptoms or signs: fever $>38^{o}$ C, urgency, frequency, dysuria, suprapubic tenderness, costovertebral angle pain or tenderness with no other recognized cause.
	Acute kidney injury	According to the KDIGO definition: Increase in serum creatinine by 27 µmol/L or more within 48 hours or increase in serum creatinine to 1.5 times baseline within 7 days.
Sepsis (other)		Strong clinical suspicion of infection but the source has not been confirmed because clinical information suggests more than one possible site, meaning two or more of the following criteria: temperature $<36^{o}$C or $>38^{o}$C, white cell count $>12 \times 10^9$/L or $< 4 \times 10^9$/L, respiratory rate >20 breaths/minute or PaCO2<4.7kPa, pulse rate >90 beats/minute.
Unplanned ITU admission		

**Surgical complications**	
Anastomotic breakdown	Anastomotic breakdown is defined as the presence of luminal contents through drain or wound site causing fever, abscess, sepsis, metabolic disturbance and/or multiple organ failure. The escape of luminal contents from the site of the anastomosis into an adjacent localised area, detected by imaging, in the absence of clinical symptoms and signs should be recorded as a subclinical leak.
Surgical site bleeding	Significant bleeding at the operation site within 72 hours after the start of surgery.
Surgical site infection	The patient has at least one of the following:<br>(a) purulent drainage from the surgical incision site<br>(b) organisms isolated from an aseptically obtained culture of fluid or tissue from the superficial incision

	(c) at least one of the following symptoms or signs of infection: pain or tenderness, localised swelling, redness or heat, the incision spontaneously dehisces or is deliberately opened by the surgeon and is culture positive or not cultured. A culture negative finding does not meet this criterion. (d) diagnosis of an incisional surgical site infection by a surgeon or attending physician.
Return to theatre	A surgical intervention for a surgical postoperative complication.
Stoma complication	A stoma problem requiring reoperation.

Table S4. KDIGO AKI classification.

Stage	Serum creatinine	Urine output
1	1.5-1.9 times baseline OR ≥ 0.3 mg/dl (≥ 26.5 μmol/L) increase	<0.5/ml/kg/h for 6-12 hours
2	2.0-2.9 times baseline	<0.5 ml/kg/h for ≥ 12 hours
3	3.0 times baseline OR increase in serum creatinine to ≥ 4 mg/dl ((≥353.6 μmol/l) OR initiation of renal replacement therapy	<0.3 ml/kg/h for ≥24 hours OR Anuria for ≥12 hours

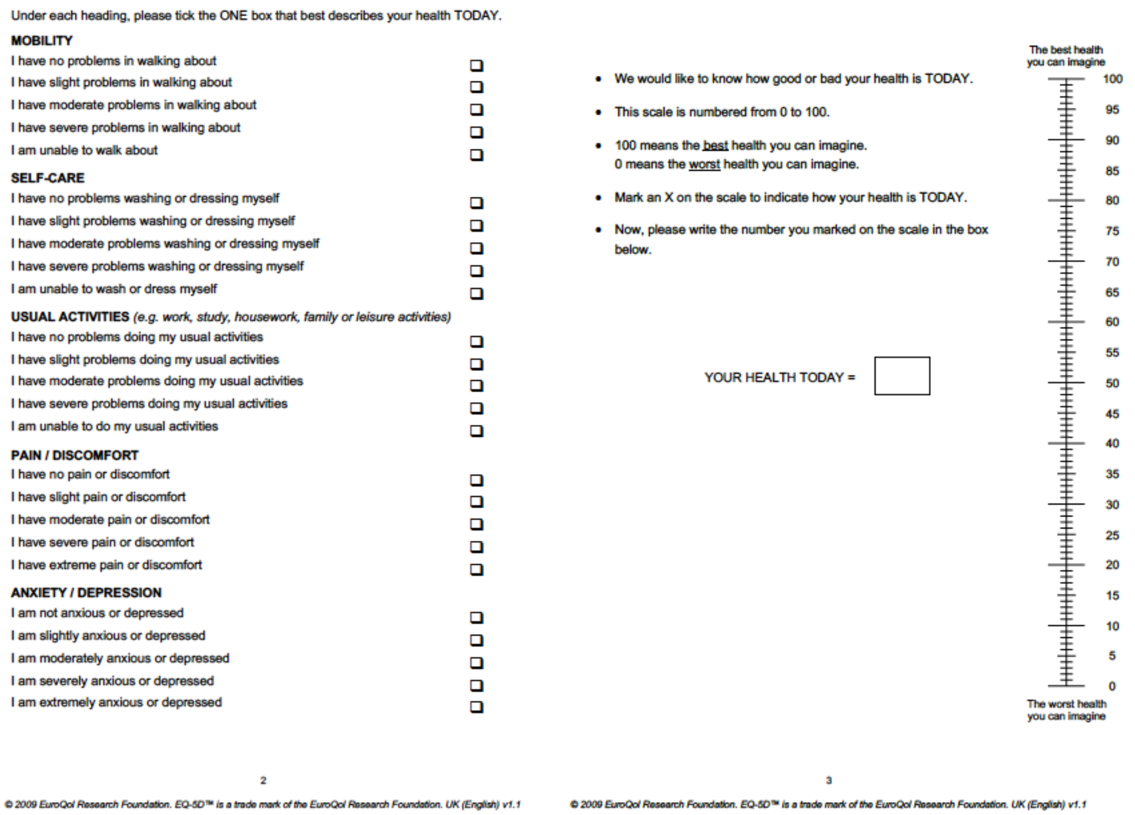
Under each heading, please tick the ONE box that best describes your health TODAY.

MOBILITY
I have no problems in walking about ❑
I have slight problems in walking about ❑
I have moderate problems in walking about ❑
I have severe problems in walking about ❑
I am unable to walk about ❑

SELF-CARE
I have no problems washing or dressing myself ❑
I have slight problems washing or dressing myself ❑
I have moderate problems washing or dressing myself ❑
I have severe problems washing or dressing myself ❑
I am unable to wash or dress myself ❑

USUAL ACTIVITIES *(e.g. work, study, housework, family or leisure activities)*
I have no problems doing my usual activities ❑
I have slight problems doing my usual activities ❑
I have moderate problems doing my usual activities ❑
I have severe problems doing my usual activities ❑
I am unable to do my usual activities ❑

PAIN / DISCOMFORT
I have no pain or discomfort ❑
I have slight pain or discomfort ❑
I have moderate pain or discomfort ❑
I have severe pain or discomfort ❑
I have extreme pain or discomfort ❑

ANXIETY / DEPRESSION
I am not anxious or depressed ❑
I am slightly anxious or depressed ❑
I am moderately anxious or depressed ❑
I am severely anxious or depressed ❑
I am extremely anxious or depressed ❑

2

© 2009 EuroQol Research Foundation. EQ-5D™ is a trade mark of the EuroQol Research Foundation. UK (English) v1.1

- We would like to know how good or bad your health is TODAY.
- This scale is numbered from 0 to 100.
- 100 means the best health you can imagine. 0 means the worst health you can imagine.
- Mark an X on the scale to indicate how your health is TODAY.
- Now, please write the number you marked on the scale in the box below.

YOUR HEALTH TODAY =

The best health you can imagine
100 95 90 85 80 75 70 65 60 55 50 45 40 35 30 25 20 15 10 5 0
The worst health you can imagine

3

© 2009 EuroQol Research Foundation. EQ-5D™ is a trade mark of the EuroQol Research Foundation. UK (English) v1.1

Figure S1. EQ-5D-5L quality of life questionnaire.

Table S5. Reasons for exclusion from the RIPCa study.

Reason for exclusion from the study	Participants
**Did not meet inclusion criteria**	39 (25%)
Risk<10%	35
Not cancer	4
**Presence of exclusion criterion**	15 (10%)
TIVA	6
DVT/PE	2
Neuromuscular disease	4
Emergency case	1
Sulfonylureas	1
Combined with liver surgery	1
**Declined participation**	21 (14%)
**In other study**	17 (11%)
**Other reasons** (unable to FU)	13 (8%)
